# A 17-Year Study of the Response of Populations to Different Patterns in Antlerless Proportion of Imposed Culls: Antlerless Culling Reduces Overabundant Deer Population

**DOI:** 10.3390/biology11111607

**Published:** 2022-11-03

**Authors:** Kei K. Suzuki, Yasumitsu Kuwano, Masatoshi Yasuda

**Affiliations:** 1 Kyushu Research Center, Forestry and Forest Products Research Institute, Kumamoto 860-0862, Kumamoto, Japan; 2 Institute of Agricultural and Forest Resources, Fukuoka Agriculture and Forestry Research Center, Kurume 839-0827, Fukuoka, Japan

**Keywords:** antlerless, culling, environmental damage, large herbivore, population, wildlife management

## Abstract

**Simple Summary:**

We evaluated the effectiveness of antlerless sika deer culling on sika deer population dynamics, based on the population trends in response to spatial variation of antlerless proportion in imposed culls. Deer populations tended to decrease in areas of higher antlerless culling, while they increased in areas of lower antlerless culling, suggesting that a high proportion of antlerless culling can effectively decrease the deer population. Because increasing large herbivore populations have caused environmental damage around the world, antlerless-biased culling could be a crucial measure to manage overabundant populations of herbivores.

**Abstract:**

Increasing populations of large herbivores have caused environmental damage around the world, and it is necessary to improve population management strategies. Culling is a traditional management method. Antlerless deer proportions, consisting of adult female deer and fawn in Cervidae in wildlife statistics, are directly related to population increases; thus, the culling-based removal of individuals from habitats and the removal of these antlerless individuals by game hunting and nuisance control might be effective approaches for reducing population sizes. We evaluated the effectiveness of antlerless culling on 17-year density trends in the sika deer (*Cervus nippon*) population across an area of 1175 km^2^ in Fukuoka Prefecture (Japan). In 11 out of 47 grids (area measuring 5 by 5 km), the densities of sika deer tended to decline; meanwhile, in the remaining 36 grids, the densities increased. These density trends were explained by changes in the proportion of antlerless culling, as the densities declined with increasing proportions of antlerless deer. The results affirm the theory that antlerless culling is effective in population management; it is posited that antlerless-biased culling could be a crucial measure in managing overabundant populations of herbivores, contributing to more effective conservation of forest environments.

## 1. Introduction

Increasing populations of large herbivores have caused environmental damage around the world [1,2,3]. Chronic high browsing pressure reduces the abundance and diversity of palatable plants and shifts vegetation structures toward unpalatable species [3,4]. In addition, long-term overbrowsing by herbivores has led to almost complete population increase failure of tree species and inhibited the natural regeneration of trees [2,5,6]. In these areas, even if the herbivore population declines, it is possible that ground vegetation will not increase for a long time [7,8,9]. Therefore, immediate reduction in the herbivore population to an appropriate density is required to conserve natural vegetation, and herbivore population management has become an important issue in reducing damage in large areas of Europe, North America, and Asia [2,10,11,12].

Adjusting herbivore abundance by culling is a traditional method of population control [2,10,11,12]. Although high culling pressure is predicted to accelerate population decline, sex- and age-specific culling can also affect the population dynamics by changing population structures [13,14]. For example, imposed culling of antlerless deer, such as fawns and adult females that are directly related to population increase, strongly influences population dynamics [15,16,17]. In areas where there is a high survival rate of adult females producing fawns, populations tend to maintain high densities [18]. Therefore, antlerless-biased culling is theoretically an effective way to reduce population sizes in a short time [19,20,21]. Buskirk et al. [22] noted the importance of large removal areas for female deer and long-term monitoring of population dynamics, even if it is local management in low-density areas. However, only a few studies have accounted for large removal areas and long-term monitoring [23]. To the best of our knowledge, other empirically studied successes have small spatial scale, such as a few square kilometres, for culling or population monitoring, and have followed the deer culling with observation for only a few years [24,25,26,27]. Alternatively, other empirical studies have showed a weak negative relationship with population density [28] or no effect [29].

Although these studies provided important knowledge for population management, it is necessary to determine the effectiveness of this strategy on larger spatial scales along administrative divisions for use in actual management for long periods. However, when evaluated on larger spatial scales, regional differences in culling pressure are more likely to occur and the impact of culling is stronger around areas of high culling pressure [30]. Therefore, to more accurately determine the effects of antlerless culling, an approach that considers spatial variation in abundance is needed. Collecting extensive data of spatiotemporal changes in population dynamics, along with culling data, is labour-intensive and costly, and these constraints have limited large-spatial-scale research into the effects of antlerless culling.

Such data have been collected for sika deer (*Cervus nippon*) to improve population management. Sika deer natively inhabits Eastern Asia and has been widely introduced into many parts of the world, including Europe, North America, and New Zealand [10]. In some areas, the deer populations have caused serious browsing damage to natural and anthropogenic environments [10,31]. As another problem of invasive population, hybridisation with native deer species was found in Europe [32,33,34,35]. Therefore, population management of this species is needed in these regions [10,35]. In addition, as sika deer have resulted in browsing damage on natural vegetation and plantations over a wide area on Kyushu Island, Japan [36,37], prefectural governments on the island imposed advanced deer culling to reduce the population, and sex and locations of culled deer were recorded [38]. The government of Fukuoka Prefecture on Kyushu Island has recorded these data for 17 years, and conducted an annual survey of deer density on a large spatial scale in the same period. These datasets are suitable for evaluating the effectiveness of antlerless culling on population management on a large spatial scale. Furthermore, it is necessary to cull at a rate above the natural mortality rate to reduce a population [39]; Simard et al. [29] cited high natural mortality as one reason why management by antlerless culling was not represented well. On Kyushu Island, there are no large carnivores acting as predators of sika deer and the mild climate brings little winter snowfall. Therefore, the small impact of these major mortality factors in general is an important advantage for accurately assessing the effectiveness of culling in this area. In this study, to test our hypothesis—that the higher the proportion of antlerless culling, the more the population will decline—we conducted large-spatial-scale analysis to evaluate the effectiveness of antlerless culling in reducing the population density in Fukuoka Prefecture, Kyushu Island, Japan.

## 2. Materials and Methods

### 2.1. Study Areas

**Figure 1:**
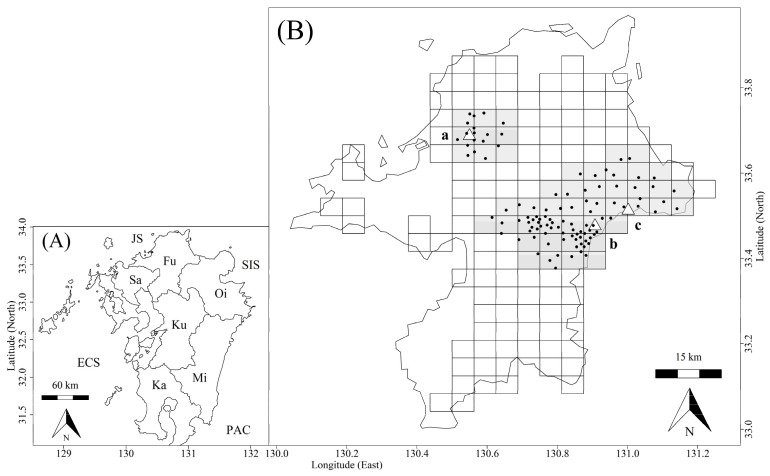
Map of Kyushu Island (**A**) and survey area in Fukuoka Prefecture (**B**). In map (**A**), Fu, Sa, Oi, Ku, Mi, and Ka indicate the Fukuoka, Saga, Oita, Kumamoto, Miyazaki, and Kagoshima prefectures, respectively. ECS, JS, and SIS indicate East China, Japan, and the Seto Inland Seas, respectively. PAC is the Pacific Ocean. In map (**B**), grids with sika deer culling (white) and with both culling and faecal pellet count survey (grey) in Fukuoka Prefecture, Japan. Plots indicate locations of faecal pellet count surveys. White triangles are locations of Mt. Inunaki-yama (a), Mt. Hiko-san (b), and Mt. Inugatake (c).

Fukuoka Prefecture was our study site, located north of Kyushu Island, Japan; it is bordered by three prefectures (Figure 1). Saga prefecture, adjacent to the west side of Fukuoka Prefecture, is devoid of sika deer. Although the deer are present in Kumamoto Prefecture, which is adjacent to the south of Fukuoka Prefecture, the density of deer on the border with Fukuoka Prefecture is low [40]. Southeast of Fukuoka Prefecture is Oita Prefecture. Deer are found in high densities along this prefectural border [40].

The forest area of Fukuoka Prefecture is approximately 2220 km^2^, accounting for approximately 45% of the total area in the prefecture. Natural forest vegetation is split into two main zones, with a boundary at an elevation of 800–1000 m, with deciduous broad-leaved forests at higher elevations and evergreen broad-leaved forests at lower elevations. In this prefecture, the climate is mild, with an average winter (December–February) temperature of approximately 6 °C and no snowfall from 2001 to 2017, according to Iizuka observatory of Japan Meteorological Agency [41].

In addition to deer, the population of large herbivores on Kyushu Island includes the Japanese serow (*Capricornis crispus*). Faeces of sika deer and serow are very similar and difficult to distinguish. However, the serow population is rapidly declining on the island, and the serow is extinct in Fukuoka Prefecture. Therefore, serow had no effect on deer density estimates in the study area.

### 2.2. Sika Deer Culling Data

The Fukuoka Prefectural Government has collected culling information, including the cull location and sex of the culled sika deer, based on reports and photos sent to the government by hunters and trappers. We obtained administrative data from 2001 to 2017. Note that “year” in this study refers to the Japanese fiscal year, running from 1 April to 31 March. The culling data covered the entire area (approximately 3700 km^2^) of the prefecture (Figure 1). In Fukuoka Prefecture, deer are culled using traps and guns. In this paper, we refer to both trappers and hunters as “hunters”. Hunters reported the locations and sex of culled deer to the Fukuoka Prefectural Government, who tallied these data for square grids of 5 km × 5 km.

Over the study period, hunters culled 73,743 individuals in 153 grids. Of these, data were missing on the culled location or sex of 2019 individuals, so they were removed from the analyses. Although sex was listed as female or male, no clear distinction was made between adults and fawns, and many fawns without antlers were included in the female category. Therefore, we treated the described females and males as antlerless and antlered, respectively.

### 2.3. Sika Deer Density Data

To estimate deer density on Kyushu Island, each prefectural government (Fukuoka, Oita, Kumamoto, Miyazaki and Kagoshima Prefectures) conducted faecal pellet counting during various seasons from 1995 to 2019. The size of each site was 50 × 200 m and the number of survey plots at each site was 110 (1 × 1 m). This method was used to calculate deer density per 1 km^2^ based on the number of faecal pellets and decay rates of the pellets according to the seasonal temperature change in each survey site [42]. A total of 1587 survey sites were set in mountain areas to cover a wide area of Kyushu Island [40]. Faecal pellets were found in 2770 of 3279 surveys, and there were no faecal pellets in the remaining 509 surveys. The number of the sites with faecal pellets was 1380. Whereas most prefectural governments conduct a survey every 4–5 years, the Fukuoka Prefectural Government has conducted the survey annually since 1995. We used data in all prefectures to estimate deer densities in Fukuoka Prefecture. Deer density data covered Mts. Inunaki-yama, Hiko-san, and Inugatake (Figure 1), with altitudes of 584, 1199, and 1131 m, respectively. Detailed survey methods and survey sites were described in our previous reports [30,40].

### 2.4. Effect of Antlerless Culling on Deer Density

To clarify the effectiveness of antlerless culling in reducing the sika deer population, we evaluated the effects of differences in the proportion of antlerless culling among square grids on trends of density changes. We constrained the analysis to only those grids that contained sites where at least one faecal pellet survey had been conducted. The analysis was performed in 47 grids, the locations of which are shown in Figure 1. The proportion of antlerless culling P in each grid, g, from 2001 to 2017, was calculated as follows:

(1) $$P_{g}=\frac{AL_{g}}{\;AL_{g}+AT_{g}}$$

where AL and AT are the numbers of antlerless and antlered individuals culled, respectively.

To clarify the density trends in each grid, we first estimated the deer density at the centre of each grid based on the densities calculated using the faecal pellet count method. In the estimation, we constructed a generalised additive model with a Tweedie distribution using the following formula:

(2) $$d_{i}=\alpha +\beta \left(\;t_{i}\right)+\gamma \left(s_{i}\right)+\varepsilon \left(s_{i},t_{i}\right)$$

where $d_{i}$, $t_{i}$, and $s_{i}$ are deer density/km^2^, year, and location (tensor production of latitude and longitude) in each survey, i, respectively. Furthermore, α is the intercept, and β, γ, and ε are fixed effects of smoothed termed year, location, and the interaction of year and location, respectively. This model included all data obtained using the faecal pellet count method on Kyushu Island. Based on this model, we estimated the density, ${\hat{d}}_{t,g}$, at the centre of each grid, g, in each year, t, as follows:

(3) $${\hat{d}}_{t,g}=\alpha +\beta \left(t\right)+\gamma \left(s_{g}\right)+\varepsilon \left(s_{g},t\right)$$

where $s_{g}$ is the location of the grid, g.

Next, we calculated the trends of sika deer density, $\tau_{{\hat{d}}_{g}}$, in each grid, g, from 2001 to 2017 when culling data were available using the Mann–Kendall trend test. Parameter τ varied between −1.0 and 1.0, with decreasing or increasing trends denoted by values closer to −1.0 or 1.0, respectively.

We evaluated the effects of antlerless culling on deer density trends using a generalised linear model (GLM) with quasi-binomial distribution. In the GLM, although we used the proportion of antlerless culling, P, as an independent variable, it is possible that culling pressure, C, and its trend, T, also affected the density trend. For example, high culling abundance, A, throughout the study period relative to low initial density, ${\hat{d}}_{t_{2001}}$, may cause a rapid population decline, independently of the proportion of antlerless culling. In addition, if culling pressure increases later in the study period, strong population decline may be seen. Therefore, to better define the effect of P by generalizing the effects of C and T, we added these effects to the model. The GLM formula with each grid used as one sample was as follows:

(4) $$\tau_{{\hat{d}}_{g}}=\rho +\varphi \left(\;P_{g}\right)+\omega \left(\;C_{g}\right)+\psi \left(\;T_{g}\right)$$

(5) $$C_{g}=\frac{A_{g}}{{\hat{d}}_{t_{2001},g}}$$

(6) $$T_{g}=\tau \left(\frac{A_{t_{2001:2017},g}}{{\hat{d}}_{t_{2001:2017},g}}\right)$$

where ρ is the intercept and φ, ω, and ψ are fixed effects of the proportion of antlerless culling, culling pressure, and trend of culling pressure, respectively. $C_{g}$ and $A_{g}$ represent the culling pressure and total culling from 2001 to 2017 in each grid, g, respectively. The trend of culling pressure in each grid, $T_{g}$, was calculated using the Mann–Kendall trend test. We estimated coefficient, *p* values, and $\chi^{2}$ values in each independent variable using the anova function in R.

The series of analyses in this study were based on Suzuki et al. [30], and all analyses were performed in R 3.5.2 [43]. In addition, *p*-values were interpreted following Muff et al. [44].

## 3. Results

**Figure 2:**
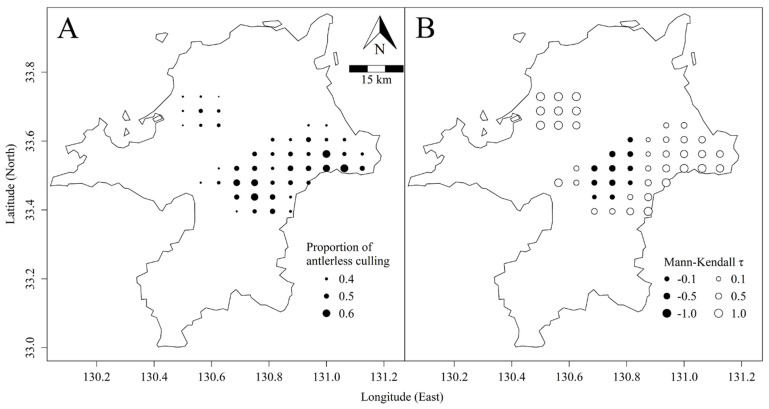
Proportions of antlerless culling of sika deer (**A**) and deer density trends (**B**) in Fukuoka Prefecture, Japan.

In total, 65,231 sika deer were culled in the 47 target grids, accounting for approximately 91% of the total cull in Fukuoka Prefecture during the study period. The proportion of antlerless culling, P, in the 47 grids was 0.504, and $P_{g}$ varied from 0.317 to 0.627. High $P_{g}$ was distributed west of Mt. Hiko-san and around Mt. Inugatake. In contrast, $P_{g}$ was relatively low on Mt. Inunaki-yama (Figure 2A).

There were regional differences in sika deer density trends. The density tended to decline in 11 grids located on the west of Mt. Hiko-san (Figure 2B). In contrast, the remaining 36 grids showed increasing trends. On Mt. Inunaki-yama especially the densities strongly increased in all grids. In addition, increasing trends were shown in some grids within the area between Mt. Hiko-san and Mt. Inugatake.

**Figure 3:**
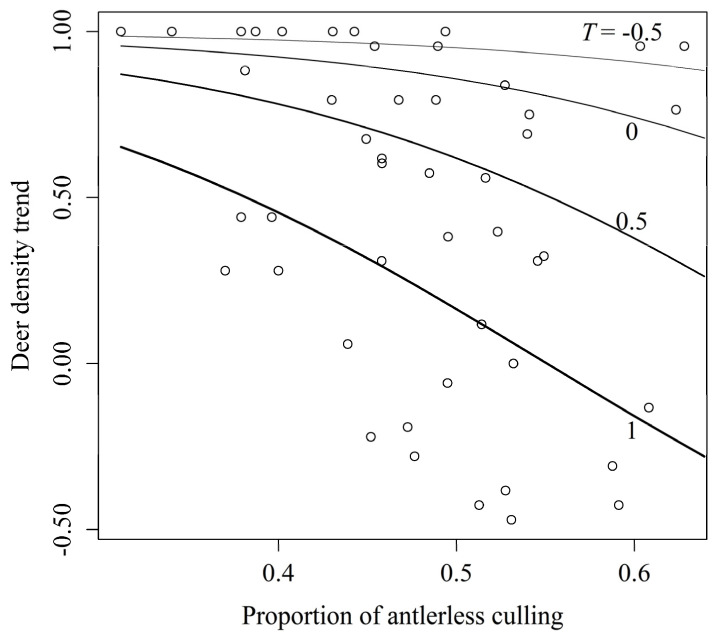
Effects of antlerless culling on sika deer density trends in Fukuoka Prefecture, Japan. Four lines indicate difference of trend of culling pressure, *T*, and values in the figure are *T*. Plots show measured values.

The GLM provided moderate evidence that P had an effect on density trends (Table 1). The density tended to decline with increasing P (Figure 3). T also effected the density trend (Table 1), and the density tended to decrease as culling pressure increased later in the study period (Figure 3).

## 4. Discussion

This study covered large spatiotemporal extents in 47 study grids (1175 km^2^) over a period of 17 years, and indicated the effectiveness of antlerless culling in population management of sika deer. The grids used for analyses were in areas with high deer densities [37,40]; in fact, 91% of the culling during the study period was within these grids. We showed previously that the proportion of antlerless deer among culled sika deer increased with increasing deer population density [38], and it is considered that proportion of antlerless deer relatively high in our study area. However, the proportion in the present study varied from 0.317 to 0.627. We considered this variation to have been due to differences in culling methods. Sika deer populations on Kyushu Island were threatened by overharvesting for venison and hides from the late 19th century to the early 20th century. In 1948, hunting of female deer was prohibited in Japan to allow the populations to recover. In 1996, female deer were again allowed to be culled legally, but as a remnant of the former restriction, culling continued to show a high proportion of antlered individuals even after lifting the prohibition against culling of females in Fukuoka Prefecture [45]. Unlike trapping, in which individuals are caught at random, gun hunting can target specific individuals. Therefore, it is likely that gun hunting was the predominant method in the grids with a low antlerless proportion in this study.

However, despite the high proportion of antlerless culling in three grids, the densities showed a strong increasing trend (Figure 3). These grids were located around Mt. Inugatake (Figure 2). This area is adjacent to neighbouring Oita Prefecture and is close to areas with relatively high deer density in Oita Prefecture [40]. Animals often move across multiple environments to track resources [46,47,48], and this movement should be considered in management planning [49]. Simard et al. [29] reported little effect of antlerless culling in a white-tailed deer (*Odocoileus virginianus*) population and cited the recolonization of surrounding deer as a potential cause. Accounting for this movement could lead to more accurate analytical results because sika deer dispersal from neighbouring prefectures was not considered in our analysis.

Unlike our study, weak or no effectiveness of antlerless culling was shown in the management of white-tailed deer populations [28,29]. This difference may be due to the unique environments of our study area. Herbivore density is usually regulated by both top–down and bottom–up effects [50,51,52]. As a major carnivorous mammal, the wolf (*Canis lupus*) has been absent for over 100 years on Kyushu Island; therefore, culling can be viewed as the only top–down effect. In contrast, bottom–up effects were not taken into consideration in the analysis. We based our analysis on administrative data and did not use an experimental approach for data collection. Therefore, no detailed environmental data were available for the study area, and we could not include environmental effects in the analysis. However, we considered there to be few bottom–up effects on Kyushu Island. Some studies have shown low mortality among adult female sika deer [18,53]. Although the severity of winter conditions—for example, insufficient food supply due to snow cover—is an important mortality factor for large herbivores [54,55,56], even a high-density sika deer population in the cold, snowy region of Hokkaido, Northern Japan, showed ≥84% female winter survival rates [18,53]. Although data on winter survival rate were not available for our study area, mortality due to food shortages in winter would be lower than in Hokkaido because of the lack of snow and mild climate.

In this study, density declines were shown in only 11 grids, while the remaining 36 grids showed an increasing trend. However, this does not negate the effectiveness of antlerless culling for population decline. As shown in some areas in this study, aggressive hunting of antlered deer is still common, such as trophy hunting [57,58]. In contrast, there are few positive reasons for hunters to hunt antlerless deer other than population management. In the future, hunters should be made aware of antlerless culling as a management tool [59] and it is necessary to present more efficient methods of antlerless culling. For example, peers in hunting groups affect the decision to participate in antlerless culling among other members [60]. In addition, improved hunter satisfaction is necessary to promote antlerless culling [61]. In Japan, the culling of sika deer is financially rewarded. It may be useful to create a disparity in the reward between male and female deer to encourage hunting groups to take part in antlerless culling.

Additionally, in this study, the increase in culling pressure was an important factor for population decline. Although this variable was needed as a covariate to correctly evaluate the effect of antlerless culling, this is not essential in the solution for population management. The years of high culling pressure were concentrated later in the study periods, which appears to have resulted in a declining trend in the population.

## 5. Conclusions

Although it has been suggested that antlerless culling is theoretically effective for deer population management [19,20,21], there are few empirical studies evidencing this hypothesis [23]. Furthermore, recent studies suggesting effective management emphasised the importance of an appropriate spatial scale and the size of the management unit [62,63]. Therefore, our study represents an important demonstration of this theory on a large spatial scale, which is more similar in size to the actual management unit of sika deer [30]. As population decline with culling can decrease browsing damage [30] and deer exclusion is effective in natural visitation restoration [64], it is hoped that antlerless culling will contribute to more effective conservation of forest environments.

Furthermore, although culling is a traditional method for population management of large herbivores [2,10,11,12], there is a need for scientific evidence of its effectiveness [65] as management by culling may not be successful for a variety of reasons [66]. We have attempted to bridge this gap between theory and practice. In addition, in this study, density trends were explained by the proportion of antlerless culling and not simply by culling pressure itself. We recommend considering the effects of individual attributes to evaluate the effectiveness of management.

## Figures and Tables

**Table 1:** Number of sites and surveys of the faecal pellet count in each prefecture.

Variables	Coefficient	SE	$\chi^{2}$	p
ρ	5.816			
φ	−6.621	3.094	1.605	0.028
ω	0.002	0.004	0.203	0.435
ψ	−2.820	1.155	2.694	0.004

ρ is intercept; φ, ω, and ψ are fixed effect of the proportion of antlerless culling, culling pressure, and trend of culling pressure, respectively.

## Data Availability

The data that support the findings of this study are available from Fukuoka Prefectural Government, but restrictions apply to the availability of these data, which were used under license for the current study, and are thus not publicly available. Data, however, are available from the corresponding author upon reasonable request and with permission of Fukuoka Prefectural Government.
